# Evaluation of Six Commercially Available Rapid Immunochromatographic Tests for the Diagnosis of Rabies in Brain Material

**DOI:** 10.1371/journal.pntd.0004776

**Published:** 2016-06-23

**Authors:** Elisa Eggerbauer, Paola de Benedictis, Bernd Hoffmann, Thomas C. Mettenleiter, Kore Schlottau, Ernest C. Ngoepe, Claude T. Sabeta, Conrad M. Freuling, Thomas Müller

**Affiliations:** 1 WHO Collaborating Centre for Rabies Surveillance and Research, Friedrich-Loeffler-Institut (FLI), Federal Research Institute for Animal Health, Institute of Molecular Virology and Cell Biology, Greifswald-Insel Riems, Germany; 2 FAO Reference Centre for Rabies, Instituto Zooprofilattico Sperimentale delle Venezie, Legnaro, Italy; 3 FLI, Institute of Diagnostic Virology, Greifswald-Insel Riems, Germany; 4 OIE Rabies Reference Laboratory, Agricultural Research Council, Onderstepoort Veterinary Institute, Pretoria, South Africa; Naval Medical Cesearch Center, UNITED STATES

## Abstract

Rabies is a neglected zoonotic disease that causes an estimated 60,000 human deaths annually. The main burden lies on developing countries in Asia and Africa, where surveillance and disease detection is hampered by absence of adequate laboratory facilities and/or the difficulties of submitting samples from remote areas to laboratories. Under these conditions, easy-to-use tests such as immunochromatographic assays, i.e. lateral flow devices (LFD), may increase surveillance and improve control efforts. Several LFDs for rabies diagnosis are available but, except for one, there are no data regarding their performance. Therefore, we compared six commercially available LFDs for diagnostic and analytical sensitivity, as well as their specificity and their diagnostic agreement with standard rabies diagnostic techniques using different sample sets, including experimentally infected animals and several sets of field samples. Using field samples the sensitivities ranged between 0% up to 100% depending on the LFD and the samples, while for experimentally infected animals the maximum sensitivity was 32%. Positive results in LFD could be further validated using RT-qPCR and sequencing. In summary, in our study none of the tests investigated proved to be satisfactory, although the results somewhat contradict previous studies, indicating batch to batch variation. The high number of false negative results reiterates the necessity to perform a proper test validation before being marketed and used in the field. In this respect, marketing authorization and batch release control could secure a sufficient quality for these alternative tests, which could then fulfil their potential.

## Author Summary

Despite being preventable with adequate biologicals, rabies still causes an estimated 60,000 human deaths annually. The main burden lies on developing countries in Asia and Africa, where dog rabies surveillance is hampered by laboratory confirmation of disease due to a number of reasons, including laboratory infrastructure and logistics. Lateral flow devices (LFD) may increase surveillance and improve control efforts. Several LFDs for rabies diagnosis are available but, except for one, there are no data available regarding their performance. Therefore, we compared six commercially available LFDs for diagnostic and analytical sensitivity. With sensitivities ranging from 0% up to 100% depending on the LFD and the samples, none of the tests investigated proved to be satisfactory, and the results somewhat contradict previous studies, indicating batch to batch variation. The high number of false negative results reiterates the necessity to perform a proper test validation before being marketed and used in the field. Only when sufficient quality is assured for these alternative tests, then they can fulfil their potential. In this respect, we demonstrated that positive results in LFD can be further validated and characterized using RT-qPCR and sequencing.

## Introduction

Rabies is an important zoonotic disease and exhibits the highest case fatality rate of any infectious disease in humans. Infection is usually transmitted by bites via saliva and it is invariably fatal once clinical signs develop. The etiological agents of the disease are the different lyssavirus species of the order *Mononegavirales*, family *Rhabdoviridae* [1]. The prototypical rabies virus (RABV) transmitted by dogs is responsible for an estimated 60,000 human deaths per year, especially in Asia and Africa [2,3]. The gold standard for rabies diagnosis is the fluorescence antibody test (FAT) [4], which is internationally approved by OIE and WHO. Briefly, brain tissue is fixed on slides, stained with fluorophore conjugated antibodies and examined under a fluorescence microscope. Confirmatory tests are virus isolation in cell culture (Webster and Casey, 1996) and the mouse inoculation test (Koprowski, 1996), the latter no longer being recommended by international organizations (OIE/WHO). Alternative diagnostics include various assays to detect viral RNA or antigen [5,6].

However, particularly in those countries that are most affected the lack of resources results in inadequate availability of equipment, chemicals and trained staff. Also, the maintenance of a cold chain during shipment of samples is difficult especially in tropical and subtropical countries, and hampers the use of these standard laboratory tests [7]. Unfortunately, the resulting inadequate rabies surveillance contributes to a cycle of neglect with a very limited number of laboratory confirmed human and animal rabies cases and thus an underestimation of the real impact of this neglected zoonotic disease, particularly in Africa and Asia [8]. Therefore, WHO has called for better tests for the rapid and economical diagnosis of RABV, without loss of sensitivity or specificity [2]. One approach to address this issue is the development of tests for the diagnosis of rabies that are relatively easy to perform, e.g. the direct rapid immunohistochemical test (dRIT), which was developed as an alternative to FAT using light microscopy [9]. Another approach is lateral flow devices (LFDs), also called rapid immunodiagnostic tests (RIDTs), immunodiagnostic assays or immunochromatographic strip tests that are interesting insofar as they have potential for field use. They are rapid and easy to use without the need for special training for implementation and evaluation. Another advantage is that these tests have no special storage requirements in terms of temperature, i.e. they can be shipped and stored at ambient room temperatures.

Their basic principle behind such tests is the fluid migration of a sample along a nitrocellulose membrane [10]. Gold conjugated antibodies bind to antigen in the sample and the antigen-antibody complex is then immobilized by a second antibody which is fixed on the test strip [11]. LFDs are applied in many different fields [10,12] including the diagnosis of viral human and animal diseases, e.g foot-and-mouth disease [13], avian influenza [14], Ebola virus disease [15], porcine epidemic diarrhea [16], Hepatitis C [17], and respiratory syncytial virus infection [18]. Recently, LFDs for rabies detection were developed and proof of principle studies yielded good results regarding sensitivity and specificity [19,20], raising hope of extending rabies diagnostic capacity in resource-limited settings [6,9]. Since then only one prototype LFD [19] was extensively evaluated, including its diagnostic range, indicating that the test is able to detect rabies and non RABV-lyssaviruses in field samples [21–24]. The routine use of LFDs for rabies diagnosis, however, is hampered by the lack of data regarding its sensitivity and specificity compared to standard diagnostic assays. In addition to the initially published prototype LFDs numerous other rabies LFDs are also commercially available for diagnostic use. Unfortunately, they have never been comprehensively analysed. Therefore, following WHO recommendations, six commercially available LFDs were compared in this study for their diagnostic and analytical sensitivity, as well as specificity, in comparison with FAT and PCR using a range of samples from experimentally infected animals and field samples.

## Materials and Methods

### Commercial LFD test kits

Six different commercial LFD test kits for rabies, i.e. Vet-o-test Rabies Ag (BioGen Technologies, Germany; LOT NO: AI191301), Anigen Rapid Rabies Ag Test kit (Bionote, Korea; LOT NO: 1801088), Quicking Pet Rapid Test (Quicking Biotech, China; LOT NO: G140210303), Rapid Rabies Ag Test Kit (Creative Diagnostics, USA; LOT NO: CD8921), Rabies Virus Ag Rapid test (Green Spring, China; LOT NO: 20140210), and quickVET Rabies Antigen Rapid test (Ubio, India; LOT NO: UB0131303).), were identified based on literature and internet searches and purchased. The price per test including tax and shipment varied between 3.14€ and 10.12€.

### Sensitivity and specificity

Sensitivity and specificity of the commercial LFDs were tested using three different sets of samples from already-existing collections of brain specimens, i.e no animals were used in this study.

Samples from sample set I and sample set II were obtained from the virus archive of the Friedrich-Loeffler-Institut (FLI). Sample set I comprised 51 samples from different parts of the brain of 17 raccoons experimentally infected with three virulent primary host adapted RABV-isolates from a European red fox, Eurasian dog and North American raccoon (Table 1) [25]. Sample set II contained 31 samples from different naturally infected brains, or mouse brain homogenates generated from field strains after mouse inoculation test (MIT), representing five different lyssavirus species. In addition to RABV variants of differing geographical origin, these species were European bat lyssavirus type 1 (EBLV-1), European bat lyssavirus type 2 (EBLV-2), Duvenhage virus (DUVV) and Bokeloh bat lyssavirus (BBLV), each of which was represented by at least one sample. The RABV field strains originated from North and South America, Asia and Europe (Table 2). Specificity was determined using five non infected brain homogenates. For both sample sets FAT was repeated for each sample essentially as previously described [4] using a four-plus scoring system. Additionally, brain material was subjected to real-time RT-PCR for confirmation and to determine the viral genome load. The quantification was performed essentially as described before [26]. Briefly, a synthetic artificial control encoding corresponding fragments of RABV, EBLV-1, EBLV-2, and BBLV was used to generate a standard curve with the R14 multiplex RT-PCR so that cq-values could be transformed into genome copies per μl template, i.e. 50mg of brain. For other lyssavirus species, the N-gene based pan-lyssa system was used [26]. Testing of those samples was conducted at the national reference laboratory for rabies at FLI, Germany. Sample set III comprised 20 brain samples of naturally infected animals including seven different animal species obtained from six different provinces of the Republic of South Africa (RSA) during rabies routine surveillance in 2015. These samples were tested with both FAT and the respective LFDs (Table 3). Test specificity was determined using 10 negative field samples. Testing of the African samples was conducted at the OIE reference laboratory at Onderstepoort Veterinary Institute, RSA using the same LOT number for each of the LFD kits tested.

**Table 1:** Diagnostic results of experimentally infected raccoons for different parts of the brain^#^.

Strain	Animal	Lab-ID	Material	FAT-Result	Genome copies/μl	BioGen	Bionote	Quicking	Creative Diagnostics	Green Spring	Ubio
RABV (dog Azerbaijan)	1	26052	AH	-	1.31E+05	-	-	-	+	-	-
	1	26053	C	+++	2.08E+05	-	-	-	+	-	-
	1	26054	MO	+++	1.52E+06	-	+	-	++	+	-
	2	26056	AH	++++	4.74E+04	-	-	-	+	-	-
	2	26057	C	++++	5.97E+04	-	-	-	+	-	-
	2	26058	MO	++++	3.56E+06	-	++	-	++	++	-
	3	26060	AH	-	6.60E+01	-	-	-	-	-	-
	3	26061	C	-	1.10E+05	-	-	-	-	-	-
	3	26062	MO	++	2.48E+05	-	-	-	-	-	-
	4	26064	AH	++++	1.68E+05	-	-	-	-	-	-
	4	26065	C	+++	4.67E+05	-	-	-	-	-	-
	4	26066	MO	++++	1.52E+07	-	+	-	++	+	-
	5	26068	AH	-	1.78E+04	-	-	-	-	-	-
	5	26069	C	++	9.19E+04	-	-	-	-	+	-
	5	26070	MO	+++	1.67E+06	-	-	-	-	-	-
	6	26072	AH	+++	1.40E+05	-	-	-	-	-	-
	6	26073	C	+++	4.17E+05	-	-	-	+	+	-
	6	26074	MO	++++	4.31E+06	-	+	-	++	++	-
RABV (fox Europe)	7	26076	AH	-	2.94E+01	-	-	-	-	-	-
	7	26077	C	-	7.03E+00	-	-	-	-	-	-
	7	26078	MO	-	2.26E+00	-	-	-	-	-	-
	8	26080	AH	+	7.49E+03	-	-	-	-	-	-
	8	26081	C	+++	3.58E+05	-	-	-	+	-	-
	8	26082	MO	++	2.19E+05	-	-	-	-	-	-
	9	26084	AH	+	4.80E+04	-	-	-	-	-	-
	9	26085	C	+++	1.21E+06	-	-	-	-	-	-
	9	26086	MO	++	4.52E+05	-	-	-	-	-	-
	10	26088	AH	+	7.67E+03	-	-	-	-	-	-
	10	26089	C	++	1.41E+05	-	-	-	-	-	-
	10	26090	MO	+++	2.39E+05	-	-	-	-	-	-
	11	26092	AH	+	8.28E+03	-	-	-	-	-	-
	11	26093	C	++	1.33E+05	-	-	-	-	-	-
	11	26094	MO	+++	2.25E+04	-	-	-	-	-	-
	12	26096	AH	+	4.93E+04	-	-	-	-	-	-
	12	26097	C	++++	1.13E+06	-	-	-	+	-	-
	12	26098	MO	+++	5.87E+05	-	-	-	-	-	-
RABV (raccoon USA)	13	26100	AH	+++	1.32E+03	-	-	-	+	+	-
	13	26101	C	++++	1.22E+04	-	+	-	++	++	-
	13	26102	MO	++++	2.27E+04	+	++	-	+++	+++	-
	14	26104	AH	++++	1.69E+04	-	-	-	-	-	-
	14	26105	C	+++	5.72E+03	-	-	-	-	-	-
	14	26106	MO	++++	3.35E+03	-	+	-	+	+	-
	15	26108	AH	+++	2.53E+03	-	-	-	-	-	-
	15	26109	C	+++	4.57E+04	-	-	-	-	-	-
	15	26110	MO	+++	5.21E+02	-	-	-	-	-	-
	16	26112	AH	++	3.59E+01	-	-	-	-	-	-
	16	26113	C	++	5.41E+02	-	-	-	-	-	-
	16	26114	MO	++++	7.35E+00	-	-	-	-	-	-
	17	26116	AH	+	6.35E+01	-	-	-	-	-	-
	17	26117	C	+++	4.54E+02	-	-	-	-	-	-
	17	26118	MO	++	3.35E+02	-	-	-	-	-	-

^#^: AH = Ammon's horn, C = cerebellum, MO = medulla oblongata

**Table 2:** Diagnostic results of archived field samples.

Lab-ID	Species	Year	Origin	Viral species	Lineage	Material	FAT-Result	Genome copies/μl	BioGen	Bionote	Quicking	Creative Diagnostics	Green Spring	Ubio
34202*	Dog	1985	Yugoslavia	RABV	Cosmopolitan (WE)	brain	++++	2.49E+07	-	+	+	++	++	-
13491*	Dog	1981	Ethiopia	RABV	Africa 1	brain	+++	1.33E+07	-	-	-	+	-	-
34203*	Wolf	1999	Yugoslavia	RABV	Cosmopolitan (WE)	brain	+++	1.28E+07	-	-	-	+	-	-
13099*	Dog	1974	Taiwan	RABV	South-East Asia	brain	++++	1.21E+07	-	++	-	+++	++	-
13255*	Human	1979	Chile	RABV	Cosmopolitan	brain	+++	3.44E+08	-	+++	-	+++	+++	-
8192	Fox	2003	Bosnia-Herzegovina	RABV	Cosmopolitan (WE)	brain	++++	2.70E+06	-	-	-	+	-	-
3139	Fox	1999	Germany	RABV	Cosmopolitan (WE)	brain	++++	5.83E+06	-	-	-	-	-	-
13133	Cat	1982	Nigeria	RABV	Africa 1	MP^#^	+	1.16E+06	-	+	-	++	+	-
13242	Bat	1966	South America	RABV	American bat variant	brain	++++	2.28E+07	-	-	-	+	-	-
13209	Mongoose	1980	South America	RABV	Cosmopolitan	MP^#^	++++	1.44E+07	-	-	-	+	-	-
13206	Raccoon	1981	North America	RABV	raccoon variant	MP^#^	+++-++++	9.81E+02	-	-	-	+	-	-
13200	Skunk	1981	USA	RABV	skunk variant	brain	++	1.14E+07	-	-	-	-	-	-
4131	Fox	1999	Czech Republic	RABV	Cosmopolitan (WE)	brain	+++	2.64E+06	-	-	-	++	+	-
13117	Dog	1983	Algeria	RABV	Africa 1	brain	++++	9.14E+07	-	-	-	++	+	-
4134	Fox	1999	Czech Republic	RABV	Cosmopolitan (WE)	brain	++++	1.97E+06	-	-	-	+	+	-
13056	Dog	1984	Turkey	RABV	Middle East	MP^#^	++++	1.31E+06	-	+	-	+	+	-
13112	Human	1974	Malaysia	RABV	South-East Asia	MP^#^	+++	3.64E+07	-	++	-	++	+++	-
13208	Vampire bat	1980	South America	RABV	American bat variant	MP^#^	++	1.59E+06	-	++	-	++	+	-
13015	Arctic fox	1981	Norway	RABV	Arctic	brain	++++	1.88E+06	-	+	-	-	-	-
13017	Arctic fox	1981	Norway	RABV	Arctic	brain	++	4.50E+05	-	-	-	+	++	-
16854	Fox	2007	Kosovo	RABV	Cosmopolitan (WE)	brain	+	5.78E+06	-	+	-	+	-	-
13512	-	1982	South Africa	RABV	Cosmopolitan (SAD vaccine strain)	brain	++-+++	7.18E+07	-	-	-	-	+	-
13114	Human	1974	Malaysia	RABV	South-East Asia	brain	++++	7.99E+06	-	-	-	-	+	-
13093	Camel	1994	Emirates	RABV	Cosmopolitan (ME)	brain	++	4.50E+06	-	++	-	+++	+	-
20299	Cattle	2008	Iraq	RABV	Cosmopolitan (ME)	brain	++++	1.24E+07	-	++	+	++	++	-
2498	Cat	1999	Germany	RABV	Cosmopolitan (WE)	brain	(+)	3.76E+00	-	-	-	-	-	-
10280	Sheep	2004	experimental	EBLV-1	-	brain	+++	8.50E+06	-	-	-	-	-	-
10270	Sheep	2004	experimental	EBLV-2	-	brain	+	2.13E+04	-	-	-	-	-	-
34494	Bat	2010	Germany	BBLV	-	MP^#^	++++	3.91E+07	-	+	-	+	-	-
34495	Bat	2012	Germany	BBLV	-	MP^#^	++++	3.28E+07	-	+	-	-	-	-
12861	Human	1974	South Africa	DUVV	-	brain	++++	1.04E+08	-	-	-	-	-	-
33341	Wolf	2014	Germany	NC^#^		brain	-	0.00E+00	-	-	-	-	-	-
33342	Wolf	2015	Germany	NC^#^		brain	-	0.00E+00	-	-	-	-	-	-
33343	Wolf	2016	Germany	NC^#^		brain	-	0.00E+00	-	-	-	-	-	-
33344	Wolf	2017	Germany	NC^#^		brain	-	0.00E+00	-	-	-	-	-	-
33345	Wolf	2018	Germany	NC^#^		brain	-	0.00E+00	-	-	-	-	-	-

^#^NC = negative controls, MP = mouse brain,

* = real-time RT-PCR was performed using RNA extracted from LFD-strips

**Table 3:** Diagnostic results of South African field samples.

Lab-ID	Species	Year	Origin	Virus species*	FAT-Result^#^	Genome copies/μl	BioGen	Bionote	Quicking	Creative Diagnostics	Green Spring	Ubio
06/15	Yellow mongoose	2015	Free State	RABV	+	8.43E+04	+	+	-	-	-	-
75/15	Yellow mongoose	2015	Mpumalanga	RABV	+	4.51E+07	+	+	-	+	+	-
102/15	Jackal	2015	North West	RABV	+	1.40E+08	+	+	-	+	+	-
110/15	Jackal	2015	North West	RABV	+	6.35E+07	+	+	-	+	+	-
149/15	Civet	2015	Mpumalanga	RABV	+	1.74E+08	+	+	-	+	-	-
14/15	Feline	2015	Limpopo	RABV	+	2.10E+07	+	+	-	+	+	-
15/15	Caracal	2015	Limpopo	RABV	+	1.15E+08	+	+	-	-	+	-
38/15	Feline	2015	Free State	RABV	+	1.73E+08	+	+	+	+	+	+
113/15	Hyena	2015	North West	RABV	+	1.92E+07	+	+	-	+	+	-
130/15	Jackal	2015	Limpopo	RABV	+	1.27E+08	+	+	-	+	-	-
36/15	Bovine	2015	Limpopo	RABV	+	4.90E+08	+	+	+	+	+	+
56/15	Bovine	2015	Free State	RABV	+	3.00E+05	+	+	-	+	+	-
139/15	Bovine	2015	Free State	RABV	+	6.68E+04	+	+	-	+	+	-
146/15	Bovine	2015	North West	RABV	+	1.79E+08	+	+	-	+	+	-
153/15	Bovine	2015	North West	RABV	+	1.00E+08	+	+	+	+	+	-
41/15	Canine	2015	Limpopo	RABV	+	5.56E+07	-	+	-	+	+	-
42/15	Canine	2015	Mpumalanga	RABV	+	7.50E+06	-	+	+	-	-	-
55/15	Canine	2015	Free State	RABV	+	3.06E+06	-	+	-	-	+	-
66/15	Wild dog	2015	North West	RABV	+	2.39E+07	+	+	-	+	+	-
125/15	Canine	2015	North West	RABV	+	2.69E+07	+	+	+	+	+	-
03/15	Canine	2015	North West	NC	-	NA	-	-	-	-	-	-
17/15	Dassie	2015	Free State	NC	-	NA	-	-	-	-	-	-
20/15	Yellow mongoose	2015	Limpopo	NC	-	NA	-	-	-	-	-	-
22/15	Sable Antelope	2015	Limpopo	NC	-	NA	-	-	-	-	-	-
26/15	Honey badger	2015	Mpumalanga	NC	-	NA	-	-	-	-	-	-
54/15	Bovine	2015	Free State	NC	-	NA	-	-	-	-	-	-
63/15	Canine	2015	Western Cape	NC	-	NA	-	-	-	-	-	-
78/15	Giraffe	2015	Mpumalanga	NC	-	NA	-	-	-	-	-	-
132/15	Feline	2015	Gauteng	NC	-	NA	-	-	-	-	-	-
136/15	Bovine	2015	Limpopo	NC	-	NA	-	-	-	-	-	-

*NC = negative controls

^#^FAT and the LFDs were only regarded positive (+) or negative (-) without any scoring the intensity

For all tests a preparation of a 10% brain homogenate (in PBS) was required after which the manufacturers’ instructions were followed. Briefly, a cotton swab was inserted into the brain suspension until saturated and then placed into the buffer solution where it was thoroughly mixed. Between two and four drops of the buffer solution were then added to the sample inlet using the disposable dropper. For the Creative Diagnostics test kit, no sample buffer was provided and PBS was used instead. The readout was made 10 min afterwards, as recommended by the manufacturers. The test and control lines on the strips were separately classified by two individuals using a three-plus scoring system representing the intensity of the reaction in the test line area.

### Analytical sensitivity

To mimic low antigen content in a potential rabid brain sample (analytical sensitivity) a two-fold positive-in-negative brain homogenate dilution series was prepared. From each of those prediluted preparations different brain suspensions in buffer were again derived, i.e. neat/undiluted, 40%, 20%, and 10%. Subsequently, the produced brain suspensions of each prediluted positive brain sample were tested by mixing 100μl with 100μl of buffer and adding 100μl to the test. Additionally, brain suspensions were subjected to real-time RT-PCR to determine the viral genome load as described above.

### Virus characterization from RABV positive LFD test strips

To investigate whether further characterization of virus in LFD test strips is possible, RNA was extracted from 30 randomly selected LFD test strips which had been stored at room temperature for six weeks. A square piece of approximately 5mm length, in the area where the test line appears, was excised, and immersed in 1ml of TriZol (Invitrogen). RNA extraction was done following manufacturer’s instructions, followed by real-time RT-PCR essentially as described [27]. Exemplarily, five of the RNA samples originating from Bionote test strips were amplified using a conventional PCR assay for subsequent partial nucleoprotein sequencing [28].

### Biosafety issues

To assess the potential presence of viable virus on the LFD, each buffer solution supplied with the test kits was tested for virus inactivation. Briefly, buffer/brain suspensions were prepared from two rabies positive samples as for use on the LFDs. A volume of 0.5 ml of those suspensions was then subjected to virus isolation in cell culture using the rabies tissue culture infection test (RTCIT) [29]. Additionally, strips of all LFDs used, except the Bionote, with a positive sample were excised 10 minutes and one hour after use and added to the prepared cell suspensions for virus isolation in cell culture. Three consecutive serial passages were considered confirmative for a negative result.

## Results

### Diagnostic sensitivity and specificity

In experimentally infected raccoons from sample set I, 44 out of 51 brain samples were positive in FAT with fluorescence scores ranging between + and ++++, whereas all samples tested positive using RT-qPCR. Most of the FAT negatives comprised samples from the Ammon’s horn. The amount of RNA per sample as determined by real-time RT-PCR ranged from 2.26 up to 1.52*10^7^ mean genome copies/μl template. The lowest amount of RNA in a FAT positive sample was 7.35 mean copies/μl template. Four of the seven FAT-negative samples had RNA content of 6.60*10^1^ mean genome copies/μl template or lower. The remaining three FAT-negative samples contained more than 1.78*10^4^ mean genome copies/μl template of RNA. Generally, the strength of agreement between results obtained by individual commercial LFDs and FAT with brain samples from experimentally infected raccoons was considered to be 'poor'. Of the 44 FAT positive samples, none tested positive using the test kits of Ubio and Quicking and one sample only tested positive using BioGen (Kappa = 0.006, 95% CI: -0.007–0.020). The other test kits detected more samples, with Bionote displaying a positive result for seven samples (Kappa = 0.049; 95% CI: -0.000–0.099), Green spring for 10 (Kappa = 0.075; 95% CI: 0.007–0.143) and Creative diagnostics for 14 samples (Kappa = 0.064; 95% CI: -0.052–0.180) (Table 1). Another sample was positive with the Creative diagnostics test kit but negative using FAT, at an RNA-content of 1.31*10^5^ mean copies/μl template. The lowest amount of viral RNA in a sample that tested positive in an LFD was 1.32*10^3^ mean copies/μl template.

Of 31 field samples from sample set II 30 tested positive and one inconclusive using FAT, while all were positive by pan-lyssa real-time RT-PCR. The amount of lyssaviral RNA in the samples ranged from 9.81*10^2^ mean copies/μl template up to 3.44*10^8^ mean copies/μl template per sample excluding one sample. Here the amount of RNA was 3.76 mean copies/μl template, presenting with only unspecific fluorescence in FAT. In contrast, all FAT positive samples were negative using Ubio and BioGen (Kappa = -0.0283; 95% CI: -0.067–0.021). Quicking displayed positive results for two samples (Kappa = -0.028; 95% CI: -0.059–0.053), while Bionote and Green spring detected 13 (Kappa = 0.085; 95% CI: -0.012–0.320) and 15 (Kappa = 0.196; 95% CI: 0.004–0.387) FAT positive samples, respectively. With 21 FAT positive samples recognized (Kappa = 0.364; 95% CI: 0.094–0.633) by the Creative diagnostics test, the correlation was considered 'fair' (Table 2). No LFD displayed a positive result with the sample that showed inconclusive fluorescence in FAT. Lyssavirus species other than RABV were negative in all LFDs except for BBLV. Creative diagnostics was able to detect one and Bionote both BBLV positive samples. All LFDs displayed a negative result for the five rabies negative samples resulting in a specificity of 100%.

With field samples from South Africa (Sample set III) all LFDs displayed a negative result for the ten rabies negative samples resulting in a specificity of 100%. The correlation between results obtained by FAT and individual commercial LFDs ranged between perfect and poor. Bionote and BioGen showed the best test results. While the correlation between FAT and Bionote was perfect, it was considered 'good' for BioGen, Green spring and Creative diagnostics. Compared to FAT, BioGen displayed a positive result for 17 (Kappa = 0.791, 95% CI: 0.571–1.000) South African samples. Green spring and Creative diagnostics each recognized 16 (Kappa = 0.727; 95% CI: 0.488–0.967) RABV positive field samples. In contrast, Quicking and Ubio detected only five (Kappa = 0.182; 95% CI: 0.013 to 0.350) and two (Kappa = 0.069; 95% CI: -0.030–0.168) FAT positive samples, respectively (Table 3).

### Analytical sensitivity

All ‘spiked’ brain-suspensions were positive using FAT (+—++++) and real-time RT-PCR. The amount of RNA in brain suspensions decreased as the dilution factor increased, starting in the undiluted positive brain at 1.24*10^7^ mean genome copies/μl template and finishing with 1.60*10^5^ mean genome copies/μl template at a dilution step of 1:128, which was the highest dilution factor used. The cut-off point up to which the LFDs were able to detect the positive brain varied, as can be seen in Table 4. Many of the test results for Bionote and Ubio could not be analyzed, since the samples did not reach either the test line or the control line. Ubio did not display a single positive result.

**Table 4:** Sensitivity assessment based using various dilutions of rabid brain (Lab-ID: 20299, n.a. = non analysable).

Dilution	FAT-Result	Genome copies/μl	Brain suspension	BioGen	Bionote	Quicking	Creative Diagnostics	Green Spring	Ubio
neat	++++	1.24E+07	neat	n.a.	n.a.	n.a.	n.a.	n.a.	n.a.
			40%	+	+++	++	+++	++	n.a.
			20%	+	+++	++	+++	+++	-
			10%	-	++	+	++	++	-
1:2	++++	4.12E+06	neat	n.a.	n.a.	n.a.	n.a.	n.a.	n.a.
			40%	+	n.a.	n.a.	+	n.a.	n.a.
			20%	-	n.a.	+	+	+	-
			10%	-	+	+	+	+	n.a.
1:4	++++	3.36E+06	neat	n.a.	n.a.	n.a.	n.a.	n.a.	n.a.
			40%	-	n.a.	+	+	+	n.a.
			20%	-	n.a.	+	+	+	-
			10%	-	+	+	+	+	-
1:8	+++	2.67E+06	neat	n.a.	n.a.	n.a.	n.a.	n.a.	n.a.
			40%	-	n.a.	+	+	+	n.a.
			20%	-	n.a.	+	+	+	-
			10%	-	+	-	-	+	-
1:16	++-+++	1.52E+06	neat	n.a.	n.a.	n.a.	n.a.	n.a.	n.a.
			40%	-	n.a.	-	n.a.	+	n.a.
			20%	-	n.a.	-	-	+	n.a.
			10%	-	n.a.	-	-	-	n.a.
1:32	++	7.53E+05	neat	n.a.	n.a.	n.a.	n.a.	n.a.	n.a.
			40%	-	n.a.	-	-	+	n.a.
			20%	-	n.a.	-	-	+	n.a.
			10%	-	-	-	-	-	-
1:64	+	4.33E+05	neat	n.a.	n.a.	n.a.	n.a.	n.a.	n.a.
			40%	-	n.a.	n.a.	n.a.	+	n.a.
			20%	-	n.a.	-	-	-	-
			10%	-	-	-	-	-	-
1:128	+	1.60E+05	neat	n.a.	n.a.	n.a.	n.a.	n.a.	n.a.
			40%	-	n.a.	-	-	-	n.a.
			20%	-	n.a.	-	-	+	n.a.
			10%	-	-	-	-	-	n.a.

### Virus characterization from RABV positive LFD test strips

The real-time RT-PCR was positive for all 30 LFD test strips with Cq values ranging between 19.12 and 37.11. Partial sequencing of the N-gene was successful for two out of 5 samples tested. When comparing the Cq values derived directly from the samples with the mean Cq values from the test strips, an increase between 11.82 and 13.51 was observed.

### Biosafety

Viable virus could be detected after mixing of RABV positive samples with the buffer solutions of Quicking, Green spring and Ubio. Also, one virus isolation was positive when the buffer solution of BioGen was used, while no positive results were obtained with Bionote buffer. After 10 minutes all test strips except Quicking still contained viable virus, but after one hour only the test strip of Creative diagnostics still contained infectious virus particles.

## Discussion

Because the gold standard of rabies diagnosis, i.e. FAT requires expensive equipment e.g. a fluorescence microscope, consumables and well trained technicians to obtain high sensitivity and specificity, it is often not applied in many endemic areas. LFDs could fundamentally facilitate and enhance rabies surveillance under these settings. The first study to evaluate a commercial rabies LFD (Bionote) for sensitivity and specificity yielded good results, but the authors concluded that the LFD should only be used for research purposes until validated or authorized for use by OIE or WHO [19]. In recent years, further studies concentrated on the Bionote LFD showing its potential to detect lyssaviruses from Africa, Asia, and Europe. With this test, sensitivities compared to FAT ranged between 91% and 100% [21–24,30,31].

Here, we compared the performance of six commercially available rabies LFDs using identical sample sets. Interestingly, sensitivity varied considerably depending on the sample set used. Clearly, sensitivity of all rabies LFDs for sample set I and sample set II were generally below expectations with a poor correlation with FAT and three tests completely failing. Originating from experimentally infected raccoons (Sample set I), the reduced sensitivity can in part be explained by the fact that for animal welfare reasons animals had to be euthanized at the onset of the first clinical signs when in some parts of the brain only little or no antigen was detectable, while viral RNA was already found [25] (Table 1). Generally, the overall RNA viral load of sample set I was lower (mean: 6.49 x 10^5^, range: 2.26 to 1.52 x 10^7^) than for sample set II (mean: 2.87 x 10^7^; range: 9.81 x 10^2^–3.44 x 10^8^) and III (mean: 8.82 x 10^7^; range: 6.68 x 10^4^–4.9 x 10^8^) where field samples mostly comprised animals that had died from the disease. Nonetheless, even samples with a high antigen load tested negative using LFDs, clearly indicating that they are unsuitable.

Comparison of performance with field samples from sample set II and sample set III, the test agreement between the individual LFDs and FAT seems contradictory. For reasons that remain unknown, particularly the LFDs from Bionote, BioGen, Green spring and Creative diagnostics showed a much better test agreement with the field sample set III from South Africa. Those results were largely confirmed when the sample set III was re-tested at FLI (S4 Table).

In additions to different RABV variants, EBLV-1, EBLV-2, DUVV, and BBLV were also included in the test panel of sample set II. In previous studies, different batches of the Bionote test had demonstrated its potential to detect lyssaviruses other than RABV from Africa and Europe [21,23], so in principle it seemed possible. However, except for BBLV, which was detected by two tests, none of the non-RABV samples tested positive. Given the high diversity in lyssaviruses [32], a broad reactivity of antibodies for capture and recognition would be ideal. This should also encompass bat lyssaviruses, as a failure to recognize lyssavirus variants could result in an incomplete picture of the epidemiological situation.

Reasons for the unsatisfactory performance of the commercial rabies LFDs could be manifold. Batch-to-batch variation could be a possible explanation for the relatively low sensitivity obtained. For example, we observed a considerably lower sensitivity of the Bionote LFD batch (41.4%) compared to previous studies [21–24,30,31] including our own results from the year 2008 (S1 Table). Similar observations were made with another batch of the Bionote tested recently in Italy (S2 Table). Also, for another test (BioGen) two different batches were analyzed and while both showed sensitivities below twenty percent, a difference in sensitivity was observed (S3 and S4 Tables).

In all tests analyzed faint reactions at the test line area were observed that made a clear differentiation of positive reactions by eyesight impossible. As this could have been indicative of low concentration of rabies or lyssavirus antigen, initially those reactions were evaluated as questionable. When testing negative samples, however, those lines also occurred occasionally and were therefore considered negative. Even with the aid of photographic technology the assessment could not be improved, thus pointing to the fact that only properly visible lines should be regarded as positive. For pen-side tests to be used directly in the field the latter option is the only solution. Another disadvantage that can be noticed in sandwich assay format LFDs is that signal generation on the test line may be compromised when the concentration of target exceeds a certain critical value [33]. Here, an excess of rabies or lyssavirus antigen could be responsible for the poor or absent signal. This possibility, however, can be excluded as in our study we clearly demonstrated that the analytical sensitivity for the tested LFDs using a pre-diluted brain suspension was generally poor with higher diluent factors having a negative influence.

Another factor influencing the sensitivity of the LFDs in this study could be the manufacturer’s instruction. Although they were all very similar, for the Bionote LFD the preparation of the samples to be tested differed between the original publication [19] and others. This is partly attributable to changes in the respective leaflet over time as shown before [22] or to other modifications being applied. For instance, eight samples from the Italian sample set that initially tested negative were positive when a modified protocol, i.e. without the first dilution step, only using the vial with buffer provided by the kit, was used (S2 Table), as had been recommended for field use [34].

Generally, a weak point of all manufacturers’ instructions was that they were not very precise regarding sample preparation, in particular the amount of brain tissue to be diluted in buffer solutions. Some instructions for instance indicated correctly to collect small pieces from different brain regions, as would be recommended for FAT. Under field conditions this may cause problems. To allow comparability, in this study we prepared one 10% brain suspension of each sample which was then used for all LFDs. Alternatively, obtaining a mixed brain sample via the occipital foramen of animals using a straw [35] could be used if animals without human contact are to be tested.

Some tests also claimed that the LFD could be used to detect virus in saliva. Because of intermittent shedding of virus, saliva-based rabies diagnosis is per se inappropriate and should be discouraged [2]. We therefore omitted to test this, also because of a lack of samples from naturally or experimentally infected animals. But even when mimicking the shedding of virus in saliva using cell culture supernatant of virus propagations, e.g. CVS (10^6.3^, 10^6.5^ TCID_50_/ml), EBLV-1 (10^6.2^ TCID_50_/ml), and EBLV-2 (10^4.3^ TCID_50_/ml) using the Bionote test in addition to field samples (S1 Table) only the undiluted supernatant and for EBLV-1a 1:10 dilution could be detected (S5 Table).

One potential disadvantage of using an LFD is its simple yes-or-no answer without further characterization of the virus. Here, we have shown that viral RNA can be stored and eventually extracted from the strip using standard procedures, similar to what has been shown for other RNA-viruses, e.g. [13–16]. If samples are additionally tested this allows not only for a confirmation of the results, but also further characterization of virus isolates. We detected viral RNA using real-time RT-PCR after six weeks of storage at room temperature. Thus strips could be easily shipped by regular mail to a specialized laboratory, e.g. to a national reference laboratory or to an internationally approved laboratory. This approach was successfully applied following our recommendation in a field trial in Ndjamena, Chad [34]. Even sequencing of the partial N-gene is possible; however this was only the case for two out of five samples. This could be explained by the six week storage of the tests at room temperature, which probably led to RNA degradation. The resulting RNA fragments may have been long enough for real-time RT-PCR but not always for sequencing, where a longer RNA fragment is needed.

Even though one LFD strip was positive in RTCIT after one hour at room temperature, generally strips can be regarded as non-infectious, as a contamination of mucous membranes is highly unlikely. However, the buffer used in those test kits should contain a virus inactivating substance, as it does for Bionote, to exclude any potential infectivity.

### Conclusions

Based on the need to improve rabies surveillance in many remote endemic areas, LFDs would be one promising alternative to laboratory testing. However, with their current limitations commercially available rabies LFDs cannot be recommended for routine diagnosis and surveillance. In particular, if animals were involved in a biting incident to a human being, false negative results may induce the patient and the doctor to refrain from appropriate post-exposure prophylaxis (PEP). Although the leaflet may explain that the results of these tests are to be confirmed by a reference method, this may not be followed and given that the cost of PEP equals a high proportion of the income in developing countries, PEP may be omitted, thus causing unnecessary deaths.

Generally, the observed limited sensitivity indicates a lack of quality control. Quality control is essentially establishing adequate performance characteristics (sensitivity, specificity, negative predictive value, positive predictive value, cross reactivity, etc.) of a given test [10,12]. Thorough validation including various circulating variants of RABV and other lyssaviruses has been recommended before those tests could be relied upon and be used as an alternative for the gold standard FAT [6]. However, it should be the responsibility of the producers and not of the customers to install a rigorous quality control system before the tests are released on the market. In some countries, e.g. Germany, any test used for the detection of a notifiable animal disease needs to obtain marketing authorization. None of the tests studied would have met the requirements for this marketing authorization and thus would not be allowed to be marketed in Germany.

This study is not meant to discredit the use of LFDs for rabies diagnosis but rather to encourage producers to substantially improve and assure the quality of their products. In principle, if those tests show a high sensitivity and specificity they could be very valuable and with their advantages in e.g. speed, easiness and storage without maintaining a cold chain could help to improve rabies detection in some parts of the world.

## Supporting Information

S1 Table Diagnostic results of archived field samples tested in 2008 using the Bionote LFD (Cat.No.:RG 18–01; Lot NO.:1801029). (PDF)

S2 Table Diagnostic results of archived field samples tested in Italy using the Bionote LFD (Lot NO.: 1801077, 1801081). (PDF)

S3 Table Comparison of two batches of BioGen LFD (Batch 1: Lot NO: AI191301, Batch 2: Lot NO: AI191402) using archived field samples (MP = mouse brain). (PDF)

S4 Table Comparison of between results obtained with sample set III in the laboratories at Onderstepoort (SA) and Friedrich-Loeffler-Institut (FLI). (PDF)

S5 Table Results of tissue culture supernatant tested in 2008 using the Bionote LFD (Cat.No.:RG 18–01; Lot NO.:1801029). (PDF)
