# Supplementary material for: Evaluation of Six Commercially Available Rapid Immunochromatographic Tests for the Diagnosis of Rabies in Brain Material
Source: PLoS Negl Trop Dis. 2016 Jun 23;10(6):e0004776. doi: 10.1371/journal.pntd.0004776 (PMC4918935; doi:10.1371/journal.pntd.0004776)
Supplement: S1 Table — (PDF) [file pntd.0004776.s001.pdf]

**Supplementary Table 1: Diagnostic results of archived field samples tested in 2008 using the Bionote LFD (Cat.No.:RG 18-01; Lot NO.:1801029)**

| Lab-ID | species | year | Origin | Viral species | Lineage | Material | FAT-Result | Bionote |
|---|---|---|---|---|---|---|---|---|
| 148 | Fox | 1998 | Germany | RABV | Cosmopolitan (WE) | brain | + | + |
| 149 | Fox | 1998 | Germany | RABV | Cosmopolitan (WE) | brain | + | + |
| 150 | Fox | 1998 | Germany | RABV | Cosmopolitan (WE) | brain | + | + |
| 151 | Fox | 1998 | Germany | RABV | Cosmopolitan (WE) | brain | + | + |
| 152 | Fox | 1998 | Germany | RABV | Cosmopolitan (WE) | brain | + | + |
| 153 | Fox | 1998 | Germany | RABV | Cosmopolitan (WE) | brain | + | - |
| 154 | Fox | 1998 | Germany | RABV | Cosmopolitan (WE) | brain | + | + |
| 155 | Fox | 1998 | Germany | RABV | Cosmopolitan (WE) | brain | + | + |
| 156 | Fox | 1998 | Germany | RABV | Cosmopolitan (WE) | brain | + | - |
| 280 | Fox | 1998 | Germany | RABV | Cosmopolitan (WE) | brain | + | + |
| 281 | Fox | 1998 | Germany | RABV | Cosmopolitan (WE) | brain | + | + |
| 282 | Sheep | 1998 | Germany | RABV | Cosmopolitan (WE) | brain | + | - |
| 284 | Fox | 1998 | Germany | RABV | Cosmopolitan (WE) | brain | + | + |
| 285 | Fox | 1998 | Germany | RABV | Cosmopolitan (WE) | brain | + | + |
| 286 | Fox | 1998 | Germany | RABV | Cosmopolitan (WE) | brain | + | + |
| 287 | Fox | 1998 | Germany | RABV | Cosmopolitan (WE) | brain | + | + |
| 288 | Fox | 1998 | Germany | RABV | Cosmopolitan (WE) | brain | + | + |
| 289 | Marten | 1998 | Germany | RABV | Cosmopolitan (WE) | brain | + | + |
| 290 | Fox | 1998 | Germany | RABV | Cosmopolitan (WE) | brain | + | + |
| 291 | Fox | 1998 | Germany | RABV | Cosmopolitan (WE) | brain | + | + |
| 292 | Fox | 1998 | Germany | RABV | Cosmopolitan (WE) | brain | + | + |
| 293 | Fox | 1998 | Germany | RABV | Cosmopolitan (WE) | brain | + | + |
| 904 | Dog | 1998 | Germany | RABV | Cosmopolitan (WE) | brain | + | + |
| 1390 | Fox | 1998 | Germany | RABV | Cosmopolitan (WE) | brain | + | - |
| 1391 | Fox | 1998 | Germany | RABV | Cosmopolitan (WE) | brain | + | - |
| 1392 | Fox | 1998 | Germany | RABV | Cosmopolitan (WE) | brain | + | - |
| 5989 | Dog | 2002 | Azerbaijan | RABV | Cosmopolitan | brain | + | + |
| 16854 | Fox | 2007 | Kosovo | RABV | Cosmopolitan | brain | + | + |
| 16862 | Fox | 2007 | Kosovo | RABV | Cosmopolitan | brain | + | + |
| 17039 | Fox | 2007 | experimental | RABV | Cosmopolitan | brain | + | + |
| 17040 | Fox | 2007 | experimental | RABV | Cosmopolitan | brain | + | + |
| 6214 | Ferret | 2002 | experimental | EBLV-1 | - | brain | + | + |
| 6215 | Ferret | 2002 | experimental | EBLV-1 | - | brain | + | + |
| 6216 | Ferret | 2002 | experimental | EBLV-1 | - | brain | + | + |
| 6217 | Ferret | 2002 | experimental | EBLV-1 | - | brain | + | + |
| 10280 | Sheep | 2004 | experimental | EBLV-1 | - | brain | + | + |
| 10282 | Sheep | 2004 | experimental | EBLV-1 | - | brain | + | + |
| 10285 | Sheep | 2004 | experimental | EBLV-1 | - | brain | + | + |
| 10271 | Sheep | 2004 | experimental | EBLV-2 | - | brain | + | + |
| 10274 | Sheep | 2004 | experimental | EBLV-2 | - | brain | + | + |
| 10277 | Sheep | 2004 | experimental | EBLV-2 | - | brain | + | + |

[#]FAT and the LFDs were only regarded positive (+) or negative (-) without scoring the intensity
