# Supplementary material for: Evaluation of Six Commercially Available Rapid Immunochromatographic Tests for the Diagnosis of Rabies in Brain Material
Source: PLoS Negl Trop Dis. 2016 Jun 23;10(6):e0004776. doi: 10.1371/journal.pntd.0004776 (PMC4918935; doi:10.1371/journal.pntd.0004776)
Supplement: S2 Table — (PDF) [file pntd.0004776.s002.pdf]

Supplementary Table 2: Diagnostic results of archived field samples tested in Italy using the Bionote LFD (Lot NO.: 1801077, 1801081)

ID number	Animal species	Origin	Viral species	Lineage	FAT result	RIDT result	RTCIT	RT-PCR
303/2010	badger	Italy	RABV	Cosmopolitan (WE)	pos	neg	pos	pos
450/2010	cat	Italy	RABV	Cosmopolitan (WE)	pos	neg	pos	pos
527/2010	roe deer	Italy	RABV	Cosmopolitan (WE)	pos	pos	pos	pos
836/2010	equine	Italy	RABV	Cosmopolitan (WE)	pos	neg	pos	pos
1546/2010	cat	Italy	RABV	Cosmopolitan (WE)	pos	pos	pos	pos
2174/2010	red fox	Italy	RABV	Cosmopolitan (WE)	pos	neg	pos	pos
2176/2010	red fox	Italy	RABV	Cosmopolitan (WE)	pos	neg	pos	pos
2177/2010	red fox	Italy	RABV	Cosmopolitan (WE)	pos	pos	pos	pos
2418/2010	roe deer	Italy	RABV	Cosmopolitan (WE)	pos	pos	pos	pos
2944/2010	red fox	Italy	RABV	Cosmopolitan (WE)	pos	pos	pos	pos
3144/2010	stone marten	Italy	RABV	Cosmopolitan (WE)	pos	neg	pos	pos
6944/2009	red fox	Italy	RABV	Cosmopolitan (WE)	pos	neg*	pos	pos
7024/2009	red fox	Italy	RABV	Cosmopolitan (WE)	pos	neg	pos	pos
117/1996	human (ex-dog)	Italy (ex-Nepal)	RABV	Arctic-like 1	pos	neg*	pos	pos
3570/2011	human (ex-dog)	Italy (ex-India)	RABV	Arctic-like 1	pos	neg*	neg	pos
1920/2005	dog	Mauritania	RABV	Africa 2	pos	neg	pos	pos
2019/2006	dog	Mauritania	RABV	Africa 2	pos	neg	pos	pos
2029/2007	dog	Mauritania	RABV	Africa 2	pos	neg	pos	pos
2049/2007	goat	Mauritania	RABV	Africa 2	pos	neg	pos	pos
1916/2005	goat	Mauritania	RABV	Africa 2	pos	neg	pos	pos
2871/2009	bovine	Botswana	RABV	Cosmopolitan (Africa 1)	pos	neg*	pos	pos
4125/2009	bovine	Botswana	RABV	Cosmopolitan (Africa 1)	pos	neg	pos	pos
3580/2009	dog	Botswana	RABV	Cosmopolitan (Africa 1)	pos	pos	pos	pos
3416/2009	goat	Botswana	RABV	Cosmopolitan (Africa 1)	pos	pos	pos	pos
5980/2009	dog	Botswana	RABV	Cosmopolitan (Africa 1)	pos	pos	pos	pos
6665/2009	honey badger	Botswana	RABV	Cosmopolitan (Africa 3)	pos	neg*	pos	pos
251/2007	dog	Niger	RABV	Africa 2	pos	neg	pos	pos
252/2007	dog	Niger	RABV	Africa 2	pos	neg	pos	pos
246/2007	dog	Niger	RABV	Africa 2	pos	neg	pos	pos
247/2007	dog	Niger	RABV	Africa 2	pos	neg	pos	pos
137/2007	dog	Burkina Faso	RABV	Africa 2	pos	neg	pos	pos
70/2007	dog	Burkina Faso	RABV	Africa 2	pos	neg	pos	pos
20/2007	dog	Burkina Faso	RABV	Africa 2	pos	pos	pos	pos
19/2007	dog	Burkina Faso	RABV	Africa 2	pos	neg	pos	pos
21/2007	dog	Burkina Faso	RABV	Africa 2	pos	neg	pos	pos
37/2007	dog	Burkina Faso	RABV	Africa 2	pos	pos	pos	pos
144/2007	dog	Burkina Faso	RABV	Africa 2	pos	neg	pos	pos
124/2007	dog	Burkina Faso	RABV	Africa 2	pos	pos	pos	pos
36/2007	dog	Burkina Faso	RABV	Africa 2	pos	neg	pos	pos
139/2007	dog	Burkina Faso	RABV	Africa 2	pos	pos	pos	pos
49/2007	dog	Burkina Faso	RABV	Africa 2	pos	neg	pos	pos
28/2007	dog	Burkina Faso	RABV	Africa 2	pos	neg	pos	pos
4314/1993	badger	Italy	RABV	Cosmopolitan (WE)	pos	neg	n.e.	pos
786/1993	chamois	Italy	RABV	Cosmopolitan (WE)	pos	neg	n.e.	pos
629/1993	cat	Italy	RABV	Cosmopolitan (WE)	pos	neg	n.e.	pos
4313/1993	marten	Italy	RABV	Cosmopolitan (WE)	pos	neg	n.e.	pos
4241/1993	red fox	Italy	RABV	Cosmopolitan (WE)	pos	neg	n.e.	pos
EURL PT2012/1 (Ariana 1991)	dog	Tunisia	RABV	Cosmopolitan (Africa 1)	pos	neg	pos	pos
EURL PT2012/2 (EBL2-VLA P3)	mouse (ex M. daubentonii)	UK	EBLV-2		pos	neg	pos	pos
EURL PT2012/3 (201020958)	mouse (ex dog)	Spain	RABV	Cosmopolitan (Africa 1)	pos	pos	pos	pos
EURL PT2012/7 (GS7)	red fox	France	RABV	Cosmopolitan (WE)	pos	neg	pos	pos
EURL PT2012/8 (GS7)	red fox	France	RABV	Cosmopolitan (WE)	pos	neg	pos	pos
EURL PT2012/9 (R75)	mouse (ex E. serotinus)	Spain	EBLV-1		pos	neg	pos	pos
351/2010	bovine	Brazil	RABV	American indigenous	pos	neg*	n.e.	pos
299/2010	bovine	Brazil	RABV	American indigenous	pos	neg	n.e.	pos
134/2010	bovine	Brazil	RABV	American indigenous	pos	neg	n.e.	pos
451/2010	bovine	Brazil	RABV	American indigenous	pos	neg	n.e.	pos
87/2010	bovine	Brazil	RABV	American indigenous	pos	neg	n.e.	pos
227/2010	bovine	Brazil	RABV	American indigenous	pos	neg	n.e.	pos
251/2010	bovine	Brazil	RABV	American indigenous	pos	neg	n.e.	pos
211/2010	bovine	Brazil	RABV	American indigenous	pos	neg	n.e.	pos
9/2010	bovine	Brazil	RABV	American indigenous	pos	neg	n.e.	pos
77/2010	bovine	Brazil	RABV	American indigenous	pos	pos	n.e.	pos
158/2011	bovine	Brazil	RABV	American indigenous	pos	neg	n.e.	pos
125/2011	bovine	Brazil	RABV	American indigenous	pos	neg	n.e.	pos
285/2011	bovine	Brazil	RABV	American indigenous	pos	neg	n.e.	pos
218/2011	bovine	Brazil	RABV	American indigenous	pos	neg	n.e.	pos
101/2011	equine	Brazil	RABV	American indigenous	pos	neg	n.e.	pos
283/2011	bovine	Brazil	RABV	American indigenous	pos	neg	n.e.	pos
303/2011	bovine	Brazil	RABV	American indigenous	pos	pos	n.e.	pos
62/2011	bovine	Brazil	RABV	American indigenous	pos	neg	n.e.	pos
320/2011	bovine	Brazil	RABV	American indigenous	pos	neg	n.e.	pos
196/2011	bovine	Brazil	RABV	American indigenous	pos	neg	n.e.	pos
144/2011	bovine	Brazil	RABV	American indigenous	pos	neg	n.e.	pos
5B1/2011	kinkajou	Brazil	RABV	American indigenous	pos	neg*	pos	pos
343/2011	equine	Brazil	RABV	American indigenous	pos	neg*	n.e.	pos

* Samples resulted positive when tested with a different protocol which skipped the first dilution step
