# Supplementary material for: Evaluation of Six Commercially Available Rapid Immunochromatographic Tests for the Diagnosis of Rabies in Brain Material
Source: PLoS Negl Trop Dis. 2016 Jun 23;10(6):e0004776. doi: 10.1371/journal.pntd.0004776 (PMC4918935; doi:10.1371/journal.pntd.0004776)
Supplement: S3 Table — (PDF) [file pntd.0004776.s003.pdf]

Supplementary Table 3: Comparison of two batches of BioGen LFD (Batch 1: Lot NO: AI191301, Batch 2: Lot NO: AI191402) using archived field samples (MP=mouse brain)

Lab-ID	species	year	Origin	Viral species	Lineage	Material	FAT-Result	Batch 1	Batch 2
34202	dog	1985	Yugoslavia	RABV	Cosmopolitan (WE)	brain	++++	++	+
13491	dog	1981	Ethiopia	RABV	Africa 1	brain	+++	-	-
34203	wolf	1999	Yugoslavia	RABV	Cosmopolitan (WE)	brain	+++	-	-
13099	dog	1974	Taiwan	RABV	South-East Asia	brain	++++	+	-
13255	human	1979	Chile	RABV	Cosmopolitan	brain	+++	++	+
8192	fox	2003	Bosnia-Herzegovina	RABV	Cosmopolitan (WE)	brain	++++	-	-
3139	fox	1999	Germany	RABV	Cosmopolitan (WE)	brain	++++	-	-
13133	cat	1982	Nigeria	RABV	Africa 1	MP	+	-	-
13242	bat	1966	South America	RABV	Cosmopolitan	brain	++++	-	-
13209	mongoose	1980	South America	RABV	Cosmopolitan	MP	++++	-	-
12861	human	1974	South-Africa	DUVV	-	brain	++++	-	-
13206	raccoon	1981	North America	RABV	raccoon variant	MP	+++-++++	-	-
10280	sheep	2004	experimental	EBLV-1	-	brain	+++	-	-
13200	skunk	1981	USA	RABV	skunk variant	brain	++	-	-
13117	dog	1983	Algeria	RABV	Africa 1	brain	++++	-	-
4134	fox	1999	Czech Republic	RABV	Cosmopolitan (WE)	brain	++++	-	-
13056	dog	1984	Turkey	RABV	Middle East	brain	++++	-	-
13208	vampire bat	1980	America	RABV	American bat variant	MP	++	-	-
13015	arctic fox	1981	Norway	RABV	Arctic	brain	++++	-	-
10270	sheep	2004	experimental	EBLV-2	-	brain	+	-	-
13017	arctic fox	1981	Norway	RABV	Arctic	brain	++	-	-
16854	fox	2007	Kosovo	RABV	Cosmopolitan (WE)	brain	+	-	-
13512	-	1982	South America	RABV	Cosmopolitan (SAD vaccine strain)	brain	++-+++	-	-
13114	human	1974	Malaysia	RABV	South-East Asia	brain	++++	+	-
2498	cat	1999	Germany	RABV	Cosmopolitan (WE)	brain	(+)	-	-
34494	mouse	2010	Germany	BBLV	-	brain	+	+	-
34495	mouse	2012	Germany	BBLV	-	brain	+	-	-
