# Supplementary material for: Evaluation of Six Commercially Available Rapid Immunochromatographic Tests for the Diagnosis of Rabies in Brain Material
Source: PLoS Negl Trop Dis. 2016 Jun 23;10(6):e0004776. doi: 10.1371/journal.pntd.0004776 (PMC4918935; doi:10.1371/journal.pntd.0004776)
Supplement: S4 Table — (PDF) [file pntd.0004776.s004.pdf]

Supplementary Table 4: Comparison of between results obtained with sample set III in the laboratories at Onderstepoort (SA) and Friedrich-Loeffler-Institut (FLI)

Lab-ID	Species	Year	Origin	Virus species	Lineage	FAT-Result	BioGen		Bionote		Quicking		Green Spring		Ubio	
							SA	FLI (new batch)	SA	FLI	SA	FLI	SA	FLI	SA	FLI
06/15	Yellow mongoose	2015	Free State	RABV	nd	+	+	-	+	-	-	-	-	-	-	NA
75/15	Yellow mongoose	2015	Mpumalanga	RABV	Mongoose	+	+	-	+	+++	-	-	+	+++	-	-
102/15	Jackal	2015	North West	RABV	Canid	+	+	-	+	++	-	-	+	++	-	-
110/15	Jackal	2015	North West	RABV	nd	+	+	-	+	+	-	-	+	+++	-	-
149/15	Civet	2015	Mpumalanga	RABV	Canid	+	+	-	+	+	-	+	-	++	-	-
14/15	Feline	2015	Limpopo	RABV	Canid	+	+	-	+	+	-	-	+	+	-	-
15/15	Caracal	2015	Limpopo	RABV	Canid	+	+	-	+	+	-	-	+	+	-	+
38/15	Feline	2015	Free State	RABV	Canid	+	+	-	+	+	+	-	+	++	+	-
113/15	Canine	2015	North West	RABV	Canid	+	+	-	+	+	-	-	+	++	-	-
130/15	Jackal	2015	Limpopo	RABV	nd	+	+	-	+	+	-	-	-	++	-	-
36/15	Bovine	2015	Limpopo	RABV	Canid	+	+	+	+	++	+	+	+	++	+	-
56/15	Bovine	2015	Free State	RABV	Mongoose	+	+	-	+	+	-	-	+	++	-	-
139/15	Bovine	2015	Free State	RABV	nd	+	+	-	+	++	-	-	+	++	-	-
146/15	Bovine	2015	North West	RABV	nd	+	+	+	+	++	-	-	+	++	-	-
153/15	Bovine	2015	North West	RABV	nd	+	+	-	+	+	+	-	+	++	-	-
41/15	Canine	2015	Limpopo	RABV	Canid	+	-	-	+	+	-	-	+	+	-	-
42/15	Canine	2015	Mpumalanga	RABV	Canid	+	-	-	+	-	+	-	-	-	-	-
55/15	Canine	2015	Free State	RABV	nd	+	-	-	+	-	-	-	+	-	-	-
66/15	Canine	2015	North West	RABV	Canid	+	+	-	+	+	-	-	+	+	-	-
125/15	Canine	2015	North West	RABV	nd	+	+	-	+	+	+	-	+	+	-	-
03/15	Canine	2015	North West	NC		-	-	-	-	-	-	-	-	-	-	-
17/15	Dassie	2015	Free State	NC		-	-	-	-	-	-	-	-	-	-	-
20/15	Yellow mongoose	2015	Limpopo	NC		-	-	-	-	-	-	-	-	+	-	-
22/15	Sable Antelope	2015	Limpopo	NC		-	-	-	-	-	-	-	-	-	-	-
26/15	Honey badger	2015	Mpumalanga	NC		-	-	-	-	-	-	-	-	-	-	-
54/15	Bovine	2015	Free State	NC		-	-	-	-	-	-	-	-	-	-	-
63/15	Canine	2015	Western Cape	NC		-	-	-	-	-	-	-	-	-	-	-
78/15	Giraffe	2015	Mpumalanga	NC		-	-	-	-	-	-	-	-	-	-	-
132/15	Feline	2015	Gauteng	NC		-	-	-	-	-	-	-	-	-	-	-
136/15	Bovine	2015	Limpopo	NC		-	-	-	-	-	-	-	-	+	-	-
