# Supplementary material for: Evaluation of Six Commercially Available Rapid Immunochromatographic Tests for the Diagnosis of Rabies in Brain Material
Source: PLoS Negl Trop Dis. 2016 Jun 23;10(6):e0004776. doi: 10.1371/journal.pntd.0004776 (PMC4918935; doi:10.1371/journal.pntd.0004776)
Supplement: S5 Table — (PDF) [file pntd.0004776.s005.pdf]

Supplementary Table 5: Results of tissue culture supernatant tested in 2008 using the Bionote LFD (Cat.No.:RG 18-01; Lot NO.:1801029)

**Virus**	**Titre (TCID50/ml)**	Undiluted	1:10	1:100	1:1000	1:10000	1:100000	1:1000000
CVS	$10^{6.5}$	+	-	-	-	-	-	-
EBLV-1	$10^{6.2}$	+	+	-	-	-	-	-
EBLV-2	$10^{4.2}$	+	-	-	-	-	-	-
